# An Ancient Drug for a Modern Era: Minocycline for the Treatment of Multi-Drug-Resistant Acinetobacter baumannii

**DOI:** 10.7759/cureus.61785

**Published:** 2024-06-06

**Authors:** Punithavathi Velmurugan, Aishwarya J Ramalingam, Chitralekha Saikumar

**Affiliations:** 1 Department of Microbiology, Sree Balaji Medical College and Hospital, Bharath Institute of Higher Education and Research, Chennai, IND; 2 Department of Microbiology, Sree Balaji Medical College and Hospital, Bharath Institute of Higher education and Research, Chennai, IND

**Keywords:** acinetobacter species, acinetobacter infections, e-strip test, colistin, minocycline, multidrug resistant acinetobacter baumannii (mdr-ab)

## Abstract

Introduction: Infections caused by *Acinetobacter baumannii *are a major cause of health concerns in the hospital setting. Moreover, the presence of extreme drug resistance in *A. baumannii* has made the scenario more challenging due to limited treatment options thereby encouraging the researchers to explore the existing antimicrobial agents to combat the infections caused by them. This study focuses on the susceptibility of multi-drug-resistant *A. baumannii* (MDR-AB) strains to minocycline and also to colistin.

Methodology: A cross-sectional study was conducted from June 2022 to June 2023. One hundred isolates of​​​​​​ *A. baumannii *​​​obtained from various clinical samples were sent to Central Laboratory, Department of Microbiology, Sree Balaji Medical College and Hospital, Chrompet, Chennai, India. The antimicrobial susceptibility testing was performed according to the Clinical and Laboratory Standards Institute (CLSI) guidelines, 2022. For the standard antibiotics, the disc diffusion method was performed. For minocycline and colistin, the minimum inhibitory concentration (MIC) was determined using an epsilometer strip (E-strip) test.

Results: In this study, 100 isolates of *A. baumannii* were obtained, and 83% of the isolates were multi-drug-resistant. Among the MDR-AB, 50 (60%) were susceptible to minocycline and 40 (48%) were susceptible to colistin. Out of the 40 colistin-susceptible *A. baumannii* strains, 29 (73%) were susceptible to minocycline with a statistically significant *P*-value of <0.05. Among the 43 colistin-resistant *A. baumannii* strains, 21 (53%) were susceptible to minocycline with a statistically significant *P*-value of <0.05.

Conclusions: When taking into account the expense of treating carbapenemase-producing Gram-negative bacteria, colistin and minocycline can be used as an alternative drug as they have fewer side effects and are more affordable. Minocycline can be used as an alternative to colistin because it is feasible to convert from an injectable to an oral formulation.

## Introduction

*Acinetobacter* is a nonmotile Gram-negative coccobacillus that is most frequently found in soil, water, and sewage [1]. It was listed by the American Society of Infectious Diseases (IDSA) as one of the six most dangerous microorganisms and the second most frequent nonfermenting Gram-negative pathogen isolated from clinical samples after *Pseudomonas aeruginosa* [2]. The Centers for Disease Control and Prevention 2019 listed carbapenem-resistant *Acinetobacter baumannii* (CRAB) as an emerging threat, particularly in hospital-acquired infections (HAI) [3]. Despite a decrease in CRAB infections probably due to infection control practices and antimicrobial stewardship [4], CRAB remains a threat due to limited treatment options [5]. There are more than 30 species in the genus *Acinetobacter*, although *A. baumannii *and, to a lesser extent, *Acinetobacter* genospecies 3 and 13TU are mostly linked to nosocomial infections in the clinical environment [6].

Immunocompromised people frequently contract *A. baumannii*, especially if they had a prolonged hospital stay (>90 days). Long hospital stays are the most common risk factor for infections caused by this organism, which can also cause HAIs like catheter-associated urinary tract infections (CAUTI), ventilator-associated events (VAE), surgical site infections (SSIs), catheter-associated bloodstream infections (CLABSI), and meningitis [7]. Furthermore, multi-drug resistance (MDR) and intrinsic resistance provide a worldwide medical concern [8]. MDR is defined as resistance to at least one drug in three or more antimicrobial categories. Carbapenems, the cornerstone of treatment in the past, are no longer successful in reducing the infections caused by this organism [9].

The percentage of organisms resistant to minocycline decreased during this time, falling from 56.5% in 2003-2005 to 30.5% in 2009-2012 [10]. The resistance towards colistin (2.8% in 2006-2008 and 6.9% in 2009-2012) and carbapenems (21.0% in 2003-2005 and 47.9% in 2009-2012) have more than doubled in the previous decade. Recent studies identified the percentage of resistance to minocycline and colistin as 7.2% and 1.7% respectively [11]. In the clinical environment, minocycline has been used as a second-line drug for a variety of bacterial infections. According to recent research, minocycline has gained importance in treating infections caused by multi-drug-resistant *A. baumannii* (MDR-AB) [12,13], due to the ability to boost colistin's efficacy against MDR-AB isolates [14]. Minocycline has better pharmacokinetic activity than other tetracyclines [15]. Thus, our study was conducted to determine the susceptibility of *A. baumannii*, with special reference to minocycline.

## Materials and methods

A cross-sectional study was conducted from June 2022 to June 2023 in the Central Laboratory, Department of Microbiology at a tertiary care hospital, Chennai, South India, after obtaining Institutional Human Ethics Committee (IHEC) approval.

Inclusion criteria

Nonrepetitive MDR-AB isolated from the culture samples sent to the Central Laboratory, Department of Microbiology during the study period were included in the study.

Exclusion criteria

Repetitive samples and highly susceptible *A. baumannii* were excluded from the study.

One hundred isolates of *A. baumannii* were obtained by conventional methods from various clinical samples such as wound swabs, ear swabs, throat swabs, pus, endotracheal aspirate (ET), blood, sputum, tissue (synovial tissue and lung tissue biopsy), and urine samples. Specimens were cultured overnight at 37 °C on 5% sheep blood agar and MacConkey agar plates. Gram staining was performed on the colonies after 24 hours, which revealed the presence of Gram-negative coccobacilli by microscopy. The biochemical tests such as catalase test, oxidase test, indole test, citrate utilization test, urease production test, triple sugar iron test, mannitol motility and fermentation test, and phenylalanine deaminase test were used to confirm the identification of the organism, as per standard operating procedure [16]. The reagents and media were obtained from HiMedia, Mumbai, India. The antimicrobial susceptibility testing was performed by the Kirby-Bauer disc diffusion method [16] and interpreted according to Clinical and Laboratory Standards Institute (CLSI) guidelines, 2022. The following drugs were used: ceftazidime (30 µg), ceftriaxone (30 µg), cefotaxime (30 µg), cefepime (30 µg), piperacillin(100 µg)-tazobactam(10 µg), doxycycline (30 µg), trimethoprim (1.25 µg)-sulfamethoxazole(23.75 µg), amikacin (30 µg), gentamicin (10 µg), tobramycin (10 µg), ciprofloxacin (5 µg), levofloxacin (5 µg), imipenem (10 µg), and meropenem (10 µg) [17]. After streaking the colonies on Muller-Hinton agar, minocycline and colistin epsilometer strips (E-strip) were placed on two separate plates [16]; observed for minimum inhibitory concentration (MIC); and interpreted as per CLSI guidelines 2022 [17]. The discs and strips were obtained from HiMedia. The controls used were satisfactory (*Escherichia coli* ATCC 25922 and *Pseudomonas aeruginosa* ATCC 27853, procured from HiMedia). The automated system - Vitek-2 compact (Vitek-2 GN card) was used for further confirmation of the organism and antimicrobial susceptibility testing (bioMérieux Inc., Durham, NC).

Data were entered in Microsoft Excel. The data were analyzed using SPSS, Version 25.0 (IBM Corp., Armonk, NY), and a *P*-value < 0.05 was considered to be statistically significant. Descriptive statistics were presented in tables as frequency and percentage.

## Results

**Figure 1:**
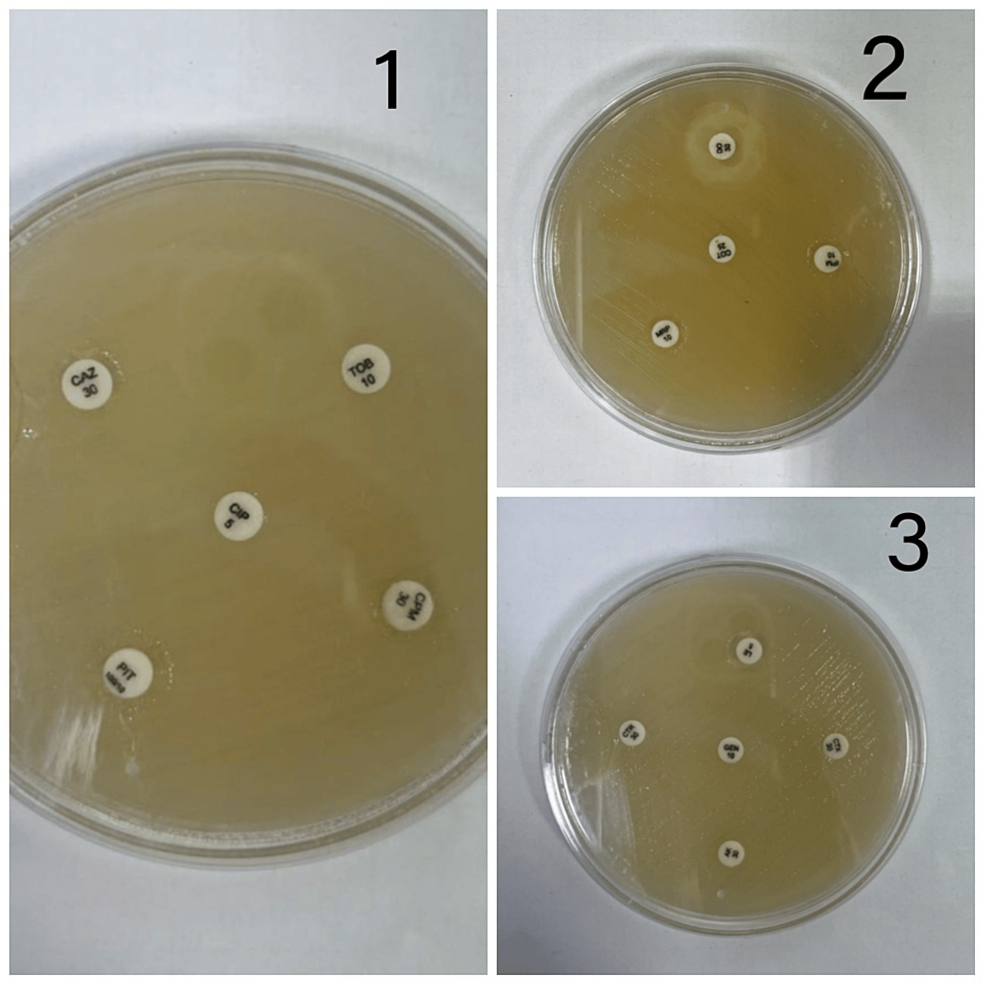
Antimicrobial susceptibility pattern of MDR-Acinetobacter baumannii by the Kirby-Bauer disc diffusion method. The following drugs were used: (1) Ceftazidime (CAZ), tobramycin (TOB), ciprofloxacin (CIP), cefepime (CPM), and piperacillin-tazobactam (PIT) (2) Doxycycline (DO), imipenem (IPM), cotrimoxazole (COT), and meropenem (MRP) (3) Ceftriaxone (CTR), levofloxacin (LE), gentamicin (GEN), cefotaxime (CTX), and amikacin (AK) MDR, multi-drug resistant

**Figure 2:**
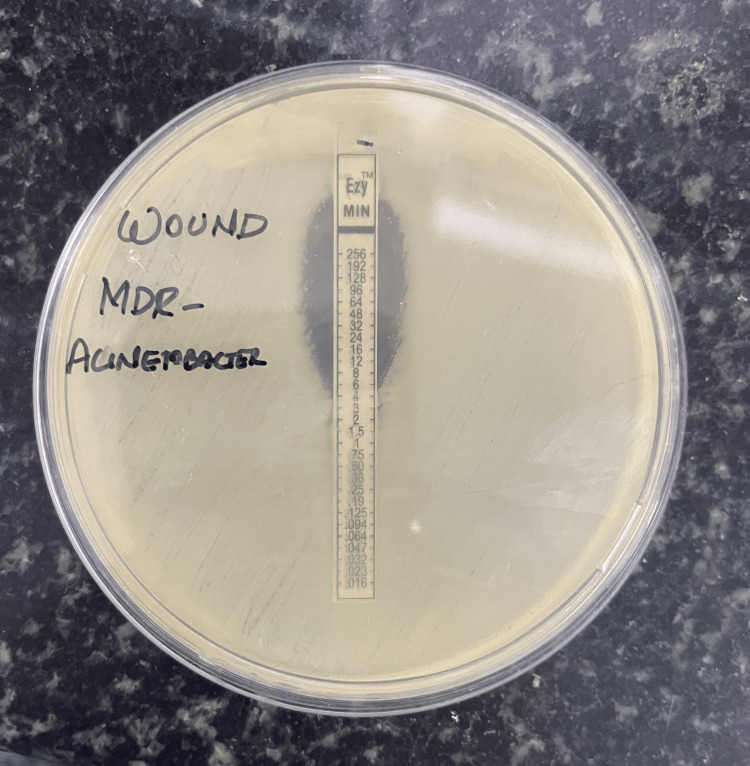
Susceptibility pattern of Acinetobacter baumannii to minocycline: epsilometer strip (E-strip) method.

**Figure 3:**
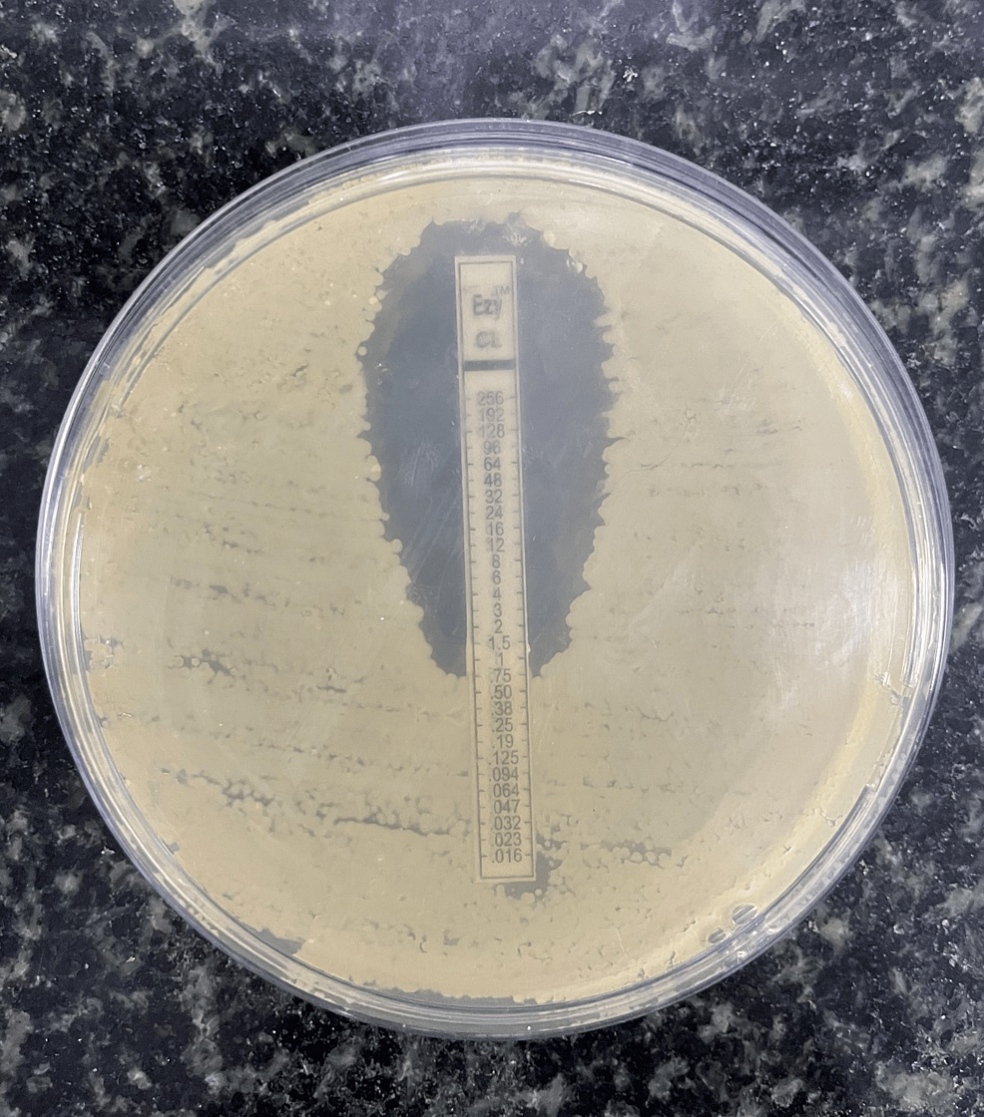
Susceptibility pattern of Acinetobacter baumannii to colistin: epsilometer strip (E-strip) method.

This study comprises 100 isolates of *A. baumannii* from a variety of clinical specimens such as wound swabs, ear swabs, throat swabs, pus, ET aspirate, blood, sputum, tissue (synovial tissue, lung tissue biopsy), and urine (Table 1). Out of the 100 *A. baumannii* isolates, 83% were MDR identified by the Kirby-Bauer disc-diffusion method (Figure 1). The antimicrobial susceptibility pattern of MDR-AB is depicted in Table 2. The antimicrobial susceptibility pattern of MDR-AB to minocycline and colistin is depicted in Tables 3-4 and Figures 2-3, respectively. 

**Table 1:** Acinetobacter baumannii among various clinical samples.

Sample	*Acinetobacter * *baumannii* (*N* = 100) (%)
Wound swab	38
Ear swab	2
Throat swab	2
Pus	12
ET	5
Blood	12
Sputum	15
Tissue (synovial tissue and lung tissue biopsy)	4
Urine	10

**Table 2:** Antimicrobial susceptibility pattern of MDR-AB. MDR-AB, multi-drug-resistant Acinetobacter baumannii

Antibiotics	Susceptible, *n* (%)	Intermediate, *n* (%)	Resistant, *n* (%)	Total
Ceftazidime	34 (41)	0	49 (59)	83
Doxycycline	42 (51)	1 (1)	40 (48)	83
Levofloxacin	51 (61)	3 (4)	29 (35)	83
Meropenem	32 (38)	1 (1)	51 (61)	83
Imipenem	30 (36)	2 (3)	51 (61)	83
Ceftriaxone	21 (25)	1 (1)	(74)	83
Cefotaxime	25 (30)	1 (1)	57 (69)	83
Piperacillin tazobactam	46 (56)	1 (1)	36 (43)	83
Cotrimoxazole	36 (43)	1 (1)	46 (56)	83
Cefepime	31 (37)	1 (1)	51 (62)	83
Gentamicin	42 (51)	2 (2)	39 (47)	83
Tobramycin	46 (55)	1 (1)	36 (44)	83
Amikacin	38 (46)	2 (2)	43 (52)	83
Ciprofloxacin	44 (53)	1 (1)	38 (46)	83

**Table 3:** Susceptibility of MDR-AB to minocycline. MDR-AB, multi-drug-resistant Acinetobacter baumannii

Drug	Susceptible,* n* (%)	Intermediate, *n* (%)	Resistant, *n* (%)	Total
Minocycline	50 (60)	20 (24)	13 (16)	83

**Table 4:** Susceptibility of MDR-AB to colistin. MDR-AB, multi-drug-resistant Acinetobacter baumannii

Drug	Susceptible, *n* (%)	Intermediate, *n* (%)	Resistant, *n* (%)	Total
Colistin	40 (48)	25 (30)	18 (22)	83

Table 5 shows the comparison of susceptibility to minocycline and colistin. Among the 40 colistin-susceptible strains, 29 (73%) showed susceptibility to minocycline, with a statistically significant *P*-value < 0.05, and also, out of the 43 colistin-resistant strains, 21 (53%) showed susceptibility to minocycline, with a statistically significant *P*-value < 0.05.

**Table 5:** Comparison of susceptibility to minocycline and colistin for MDR-AB. MDR-AB, multi-drug-resistant Acinetobacter baumannii

Drug	Minocycline susceptible, *n* (%)	Minocycline resistant, *n* (%)	Total
Colistin susceptible	29 (73)	11 (27)	40
Colistin resistant	21 (53)	22 (47)	43
Total	50	33	83

## Discussion

CRAB has become more common though carbapenems are effective treatments for MDR-AB infections. In Asia, 60% of the isolates were CRAB and pan-drug-resistant *A. baumannii*. In the United States and Europe, 65% of the isolates were identified as CRAB [18]. Numerous tigecycline resistance mechanisms in *A. baumannii* can be circumvented by minocycline. Additionally, minocycline has good in vitro action against drug-resistant *A. baumannii* and favorable pharmacokinetic and pharmacodynamic characteristics. Minocycline is considered to be an effective alternative against MDR infections, particularly in conditions where limited antimicrobials are accessible and in patients requiring prolonged hospitalization. Akers et al. demonstrated a favorable clinical outcome in such patients with minocycline in their trial in a military center against MDR *Acinetobacter* spp. [19]. Flamm et al. showed minocycline to be progressively efficient against MDR pathogens in a comparison investigation using tetracycline, doxycycline, and minocycline. 70% of MDR organisms were sensitive to minocycline in their study [20]. Greig and Scott detected a susceptibility of 80% toward minocycline among MDR *Acinetobacter* spp. [21].

Fragkou et al. found that the clinical and microbiological success rates after minocycline treatment were 72.6% and 60.2%, respectively [22]. In our study, out of 83 MDR-AB, 50 (60%) were susceptible to minocycline, 20 (24%) were intermediate, and 13 (16%) were resistant, which implies minocycline is effective against multi-drug-resistant *Acinetobacter* spp. either by oral or by intravenous (IV) route. Greig and Scott explained the potential role of IV minocycline efficacy against MDR *Acinetobacter* spp. especially when combined with a second antibacterial agent [21]. IV minocycline has been approved by the US FDA for the treatment of culture-positive MDR *Acinetobacter* species in hospitalized patients. The "Generating Antibiotic Incentives Now Act (GAIN Act)" designates its approval as a Qualified Infectious Disease Product (QIDP) [23]. Fragkou PC et al. in the systematic review of the outcome of minocycline treatment towards MDR-AB emphasized a substantial percentage of success in clinical (72.6%) and microbiological (60.2%) aspects [22]. In the study conducted by Kuo SC et al. approximately 20% of *A. baumannii* isolates were resistant to minocycline [24]. However, its therapeutic appeal has been increased by the simplicity of switching from IV to oral formulations. This is especially true for patients who are hard to treat and have resistant *Acinetobacter* infections, as the data that is now available supports the efficacy of therapy in these tough clinical situations [15].

Colistin is used as a rescue therapy for severe infections and is regarded as one of the final therapeutic alternatives for the treatment of MDR-AB infections [25,26]. Due to increasing resistance to colistin and due to its toxicity and higher cost, we can use minocycline as an alternative to colistin in most multi-drug-resistant *Acinetobacter* cases. The most commonly used therapeutic combinations for MDR-AB include carbapenems, tigecycline, minocycline, polymyxins, and daptomycin. Bowers et al. in a study, where minocycline and polymyxin B were used as a combination on pan-drug-resistant *A. baumannii* found out that the MIC of each medication significantly dropped [27]. As a result, either as a single drug or in combination with other drugs it is very effective against MDR *Acinetobacter* spp.

Limitations of the study

This study includes only bacterial isolates from the lab, and hence, patient details, clinical outcomes, and correlation were not analyzed. The comparison of various antimicrobial susceptibility methods for colistin such as the micro-broth dilution method was not performed.

## Conclusions

Choosing the right medications is still crucial in treating infections caused by multi-drug-resistant organisms such as *A. baumannii*. In certain circumstances, minocycline can be used in the place of colistin. This is notably true for patients with MDR organisms hospitalized in intensive care units, where minocycline has fewer side effects and is more affordable. When taking into account the expense of treating Gram-negative bacteria that produce carbapenemases, colistin, and minocycline can be taken as an alternative drug. A potential therapeutic niche for minocycline would be the conversion of an injectable mode to oral formulation in stable patients who need a longer course of treatment.
